# Supplementary material for: Genome-wide analysis of differential transcriptional and epigenetic variability across human immune cell types
Source: Genome Biol. 2017 Jan 26;18:18. doi: 10.1186/s13059-017-1156-8 (PMC5270224; doi:10.1186/s13059-017-1156-8)
Supplement: Additional file 1: — Supplementary figures. Supplementary document (.pdf) containing all Supplementary figures. (PDF 1165 kb) [file 13059_2017_1156_MOESM1_ESM.pdf]

Table of Contents

SUPPLEMENTARY FIGURES

Figure S1. Quality assessment of batch effect correction for RNA-seq and DNA methylation data 2
Figure S2. Quality assessment of the gene expression data set generated using RNA-seq 3
Figure S3. Quality assessment of the DNA methylation data set generated using 450K arrays................ 4
Figure S4. Robust quantification of inter-individual variability of gene expression levels....................... 5
Figure S5. Genetic determinants of hypervariable genes and CpGs in the BLUEPRINT Human Variation Panel 6
Figure S6. Sex-specific differences in gene expression across immune cells 7
Figure S7. Functionally grouped annotation network of a highly correlated network module of neutrophil-specific hypervariable genes not mediated by *cis* genetic effects 8
Figure S8. Robust quantification of inter-individual variability of DNA methylation levels.................... 9
Figure S9. Correlation of cell type-specific hypervariable CpGs with donor information...................... 10
Figure S10. Global relationship between DNA methylation variability and gene expression variability at gene promoters and bodies 11
Figure S11. Gene expression variability of surface markers corresponding to known neutrophil subpopulations 12

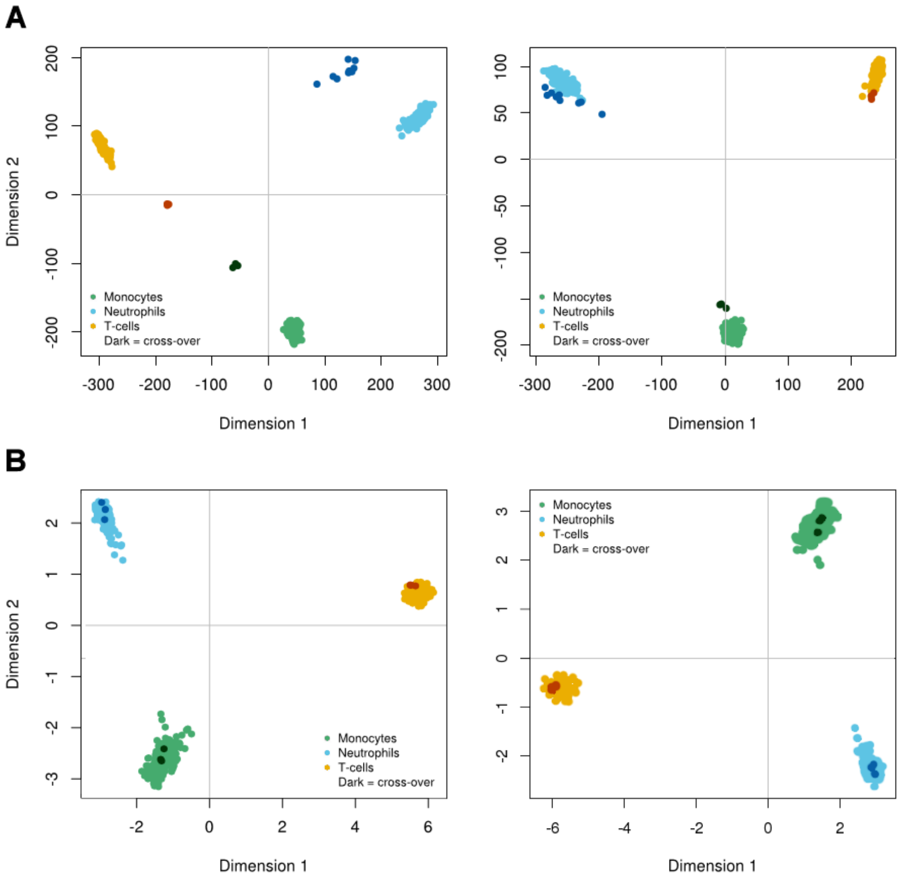

Figure S1. Quality assessment of batch effect correction for RNA-seq and DNA methylation data. Multidimensional scaling (MDS) before and after batch effect correction to assess center effects using cross-over samples. All samples generated by the Human Variation Panel (Chen et al. (2016), *Cell* 167(5), 1398-414) are included. (A) MDS of RNA-seq data before (left panel) and after (right panel) batch effect correction. (B) MDS of DNA methylation data before (left panel) and after (right panel) batch effect correction.

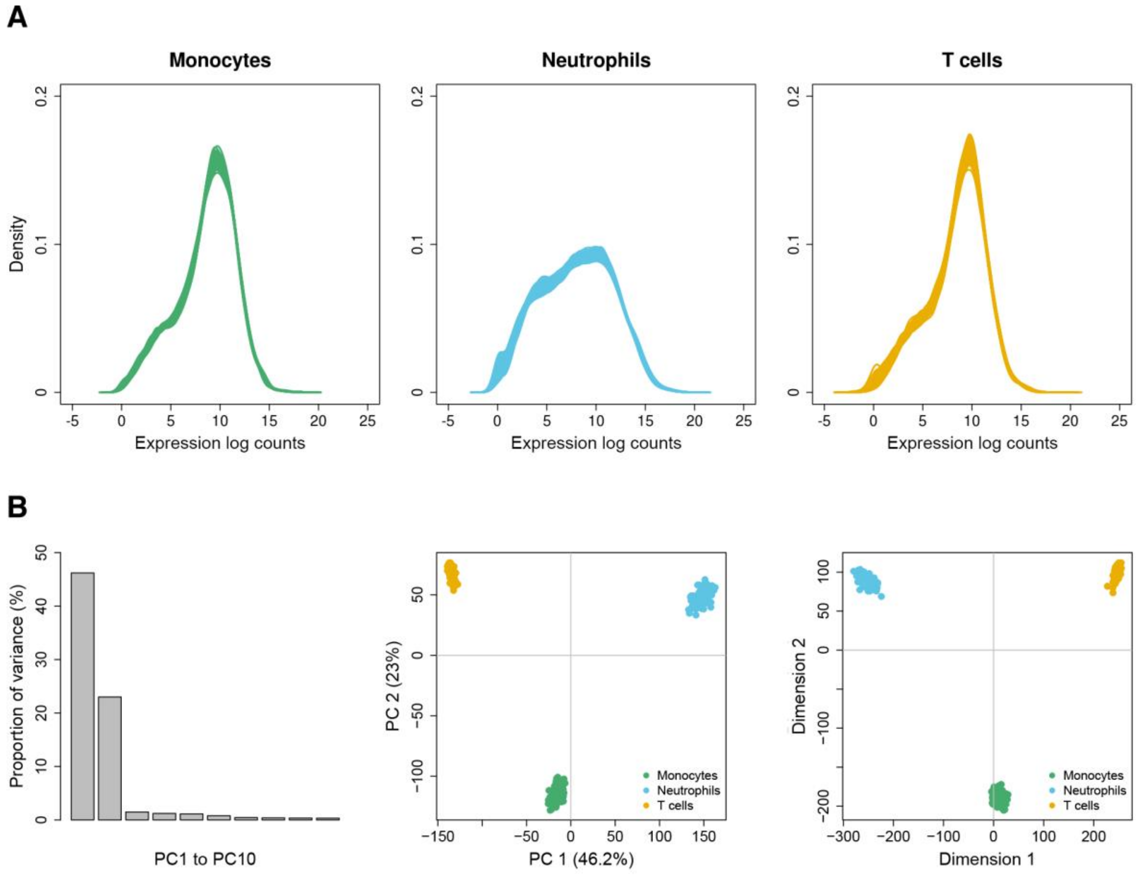

Figure S2. Quality assessment of the gene expression data set generated using RNA-seq. Quality assessment of the RNA-seq data after normalization and batch effect correction. We filtered non-protein coding genes, and genes that were not expressed in at least 50% of the samples in each of the three cell types. This resulted in a final set of 11,980 protein-coding genes. (A) Distribution of read counts (logarithmic scale) for the three profiled immune cell types. (B) Proportion of variance explained by the first ten principal components (left panel), principal component analysis (PCA; middle panel), and multidimensional scaling (MDS; right panel).

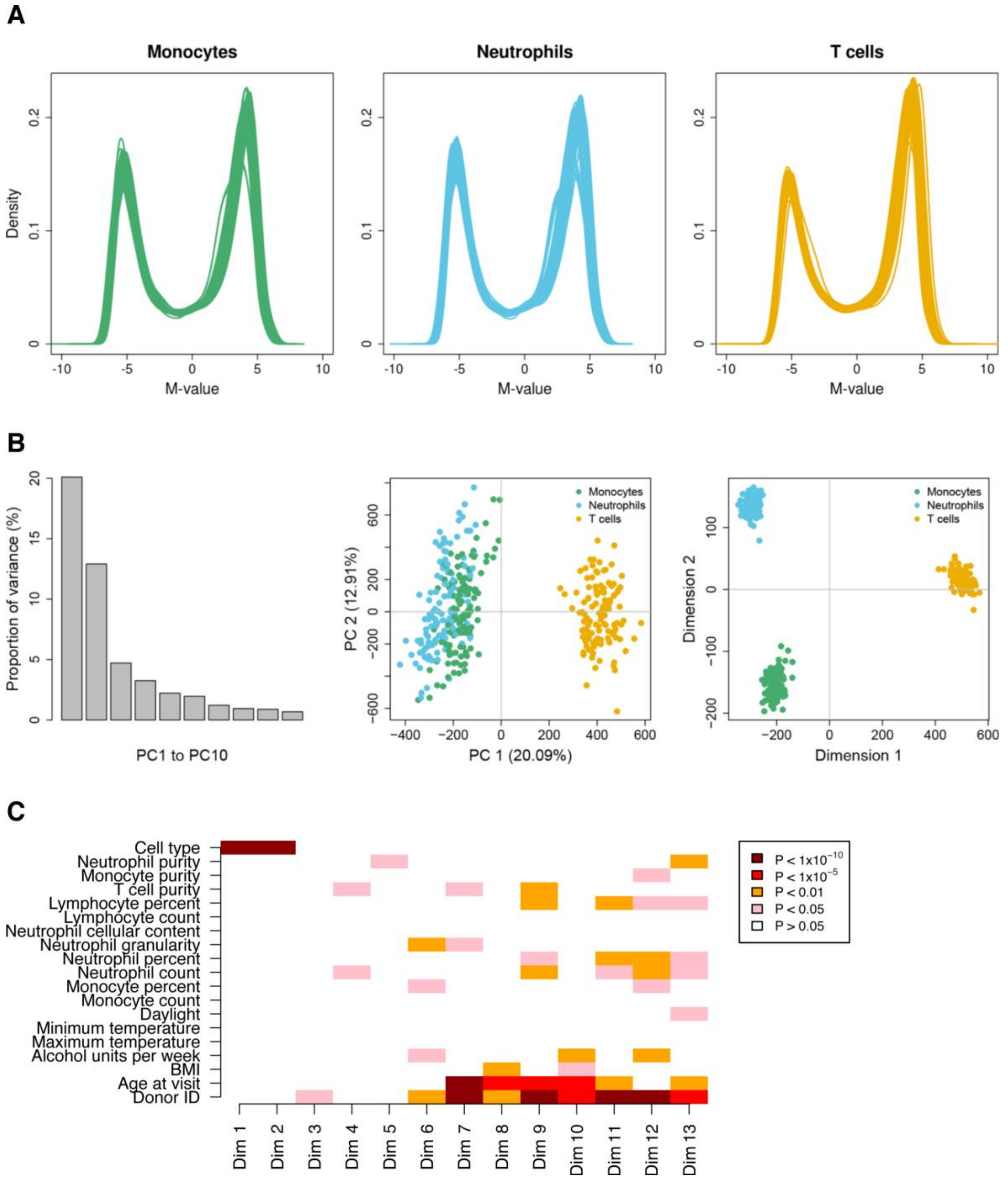

Figure S3. Quality assessment of the DNA methylation data set generated using 450K arrays. Quality assessment of the 450K array data after normalization, probe filtering, and batch effect correction. The final data set contained 440,905 CpG sites. (A) Distribution of DNA methylation M-values for all three profiled immune cell types. (B) Proportion of variance explained by the first ten principal components (left panel), PCA (middle panel), and MDS (right panel). (C) Singular value decomposition (SVD). The heatmap indicates the nature of the largest components of variation.

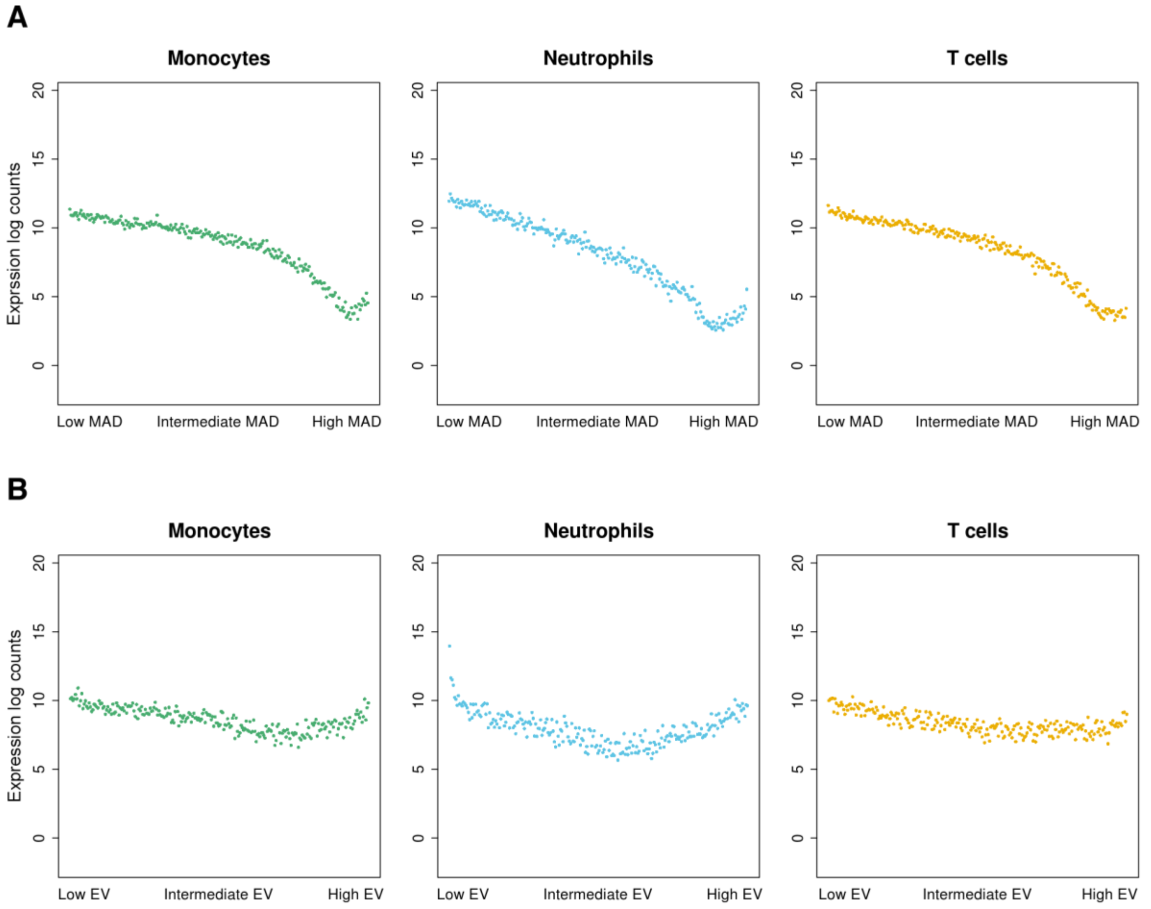

Figure S4. Robust quantification of inter-individual variability of gene expression levels. We compared two parameters that give account of gene expression variability, the median absolute deviation (MAD) and gene expression variability value (EV). First, gene-wise MAD-values were calculated. Then, these values were ordered from low to high MAD, grouped together in bins of 100 genes, and plotted against their corresponding mean gene expression levels. The ordering by MAD-values was maintained to examine whether the MAD-values are evenly distributed across gene expression levels. The same was done for EV-values. (A) RNA-seq read counts (logarithmic scale) with regards to MAD-values, ordered from low to high. (B) RNA-seq read counts with regards to EV-values. In contrast to MAD-values, EV-values showed less dependence on the mean, thus enabling unbiased quantification of gene expression variability.

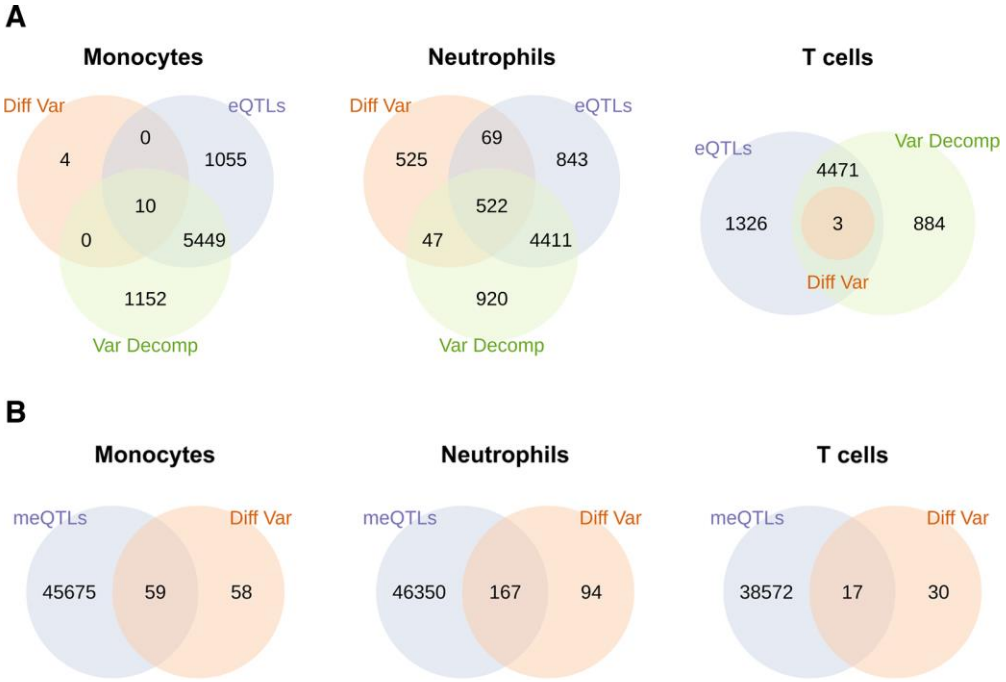

Figure S5. Genetic determinants of hypervariable genes and CpGs in the BLUEPRINT Human Variation Panel. (A) Venn diagrams showing the overlap between genes identified using the approach to assess differential variability described in this study (labeled “Diff Var”), genes associated with local genetic variants (i.e. expression QTLs, labeled “eQTLs”), and genes estimated to be under genetic control using variance decomposition modeling (labeled “Var Decomp”). (B) Venn diagrams showing the overlap between CpG sites identified to exhibit hypervariable DNA methylation levels using the approach described here (labeled “Diff Var”) and CpGs associated with *cis* genetic variants (i.e. DNA methylation QTLs, labeled “meQTLs”).

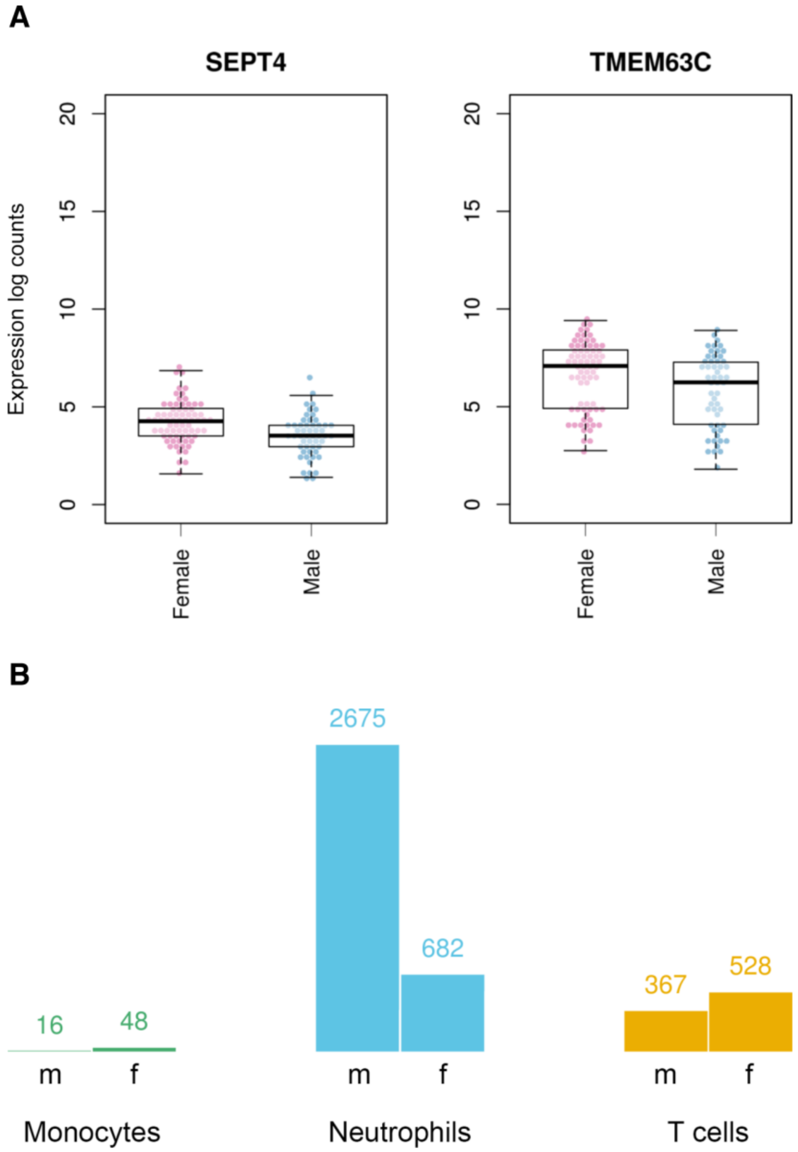

Figure S6. Sex-specific differences in gene expression across immune cells. (A) Boxplots showing the expression levels of the neutrophil-specific hypervariable genes *SEPT4* and *TMEM63C*. These two HVGs were the only ones that were found to be differently expressed between male and female donors at a log-fold change (FC) of ≥1, with $P = 6.35\times10^{-5}$ (FC = 1.04) and $P = 5.66\times10^{-4}$ (FC = 1.08), respectively. Data points represent the expression values of the indicated gene in one individual. (B) Barplots showing the number of genes with significantly higher expression levels in females (labeled "f") and males (labeled "m") for each cell type.

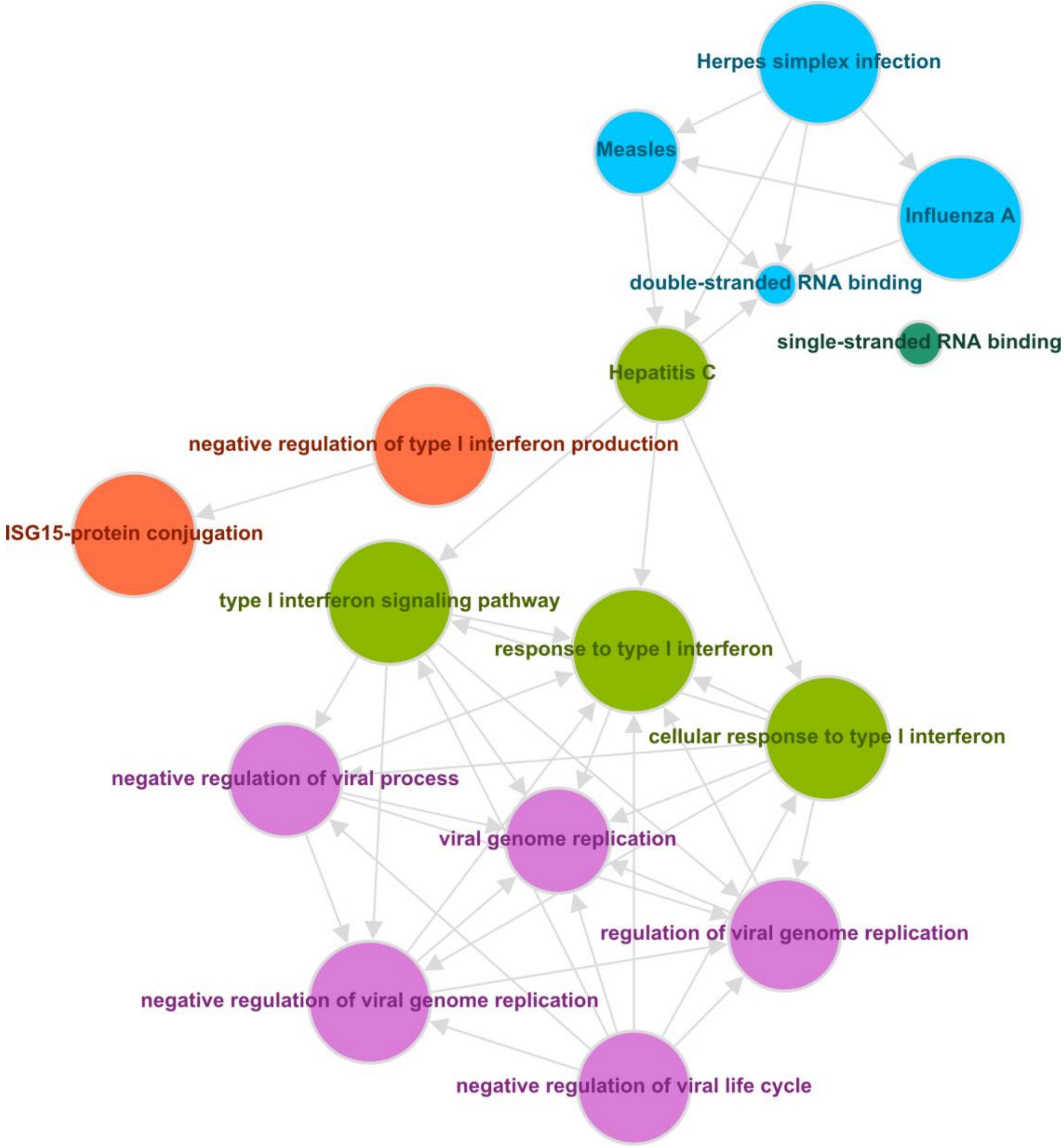

Figure S7. Functionally grouped annotation network of a highly correlated network module of neutrophil-specific hypervariable genes not mediated by *cis* genetic effects. Gene ontology and KEGG pathway enrichments of the genes contained in the red network module of Figure 3. Node sizes are determined by BH-corrected *P*-values, with bigger nodes indicating smaller *P*-values. Functionally related terms are shown in the same color. Connections between nodes reflect the relationship between the significant terms based on the similarity of their associated genes.

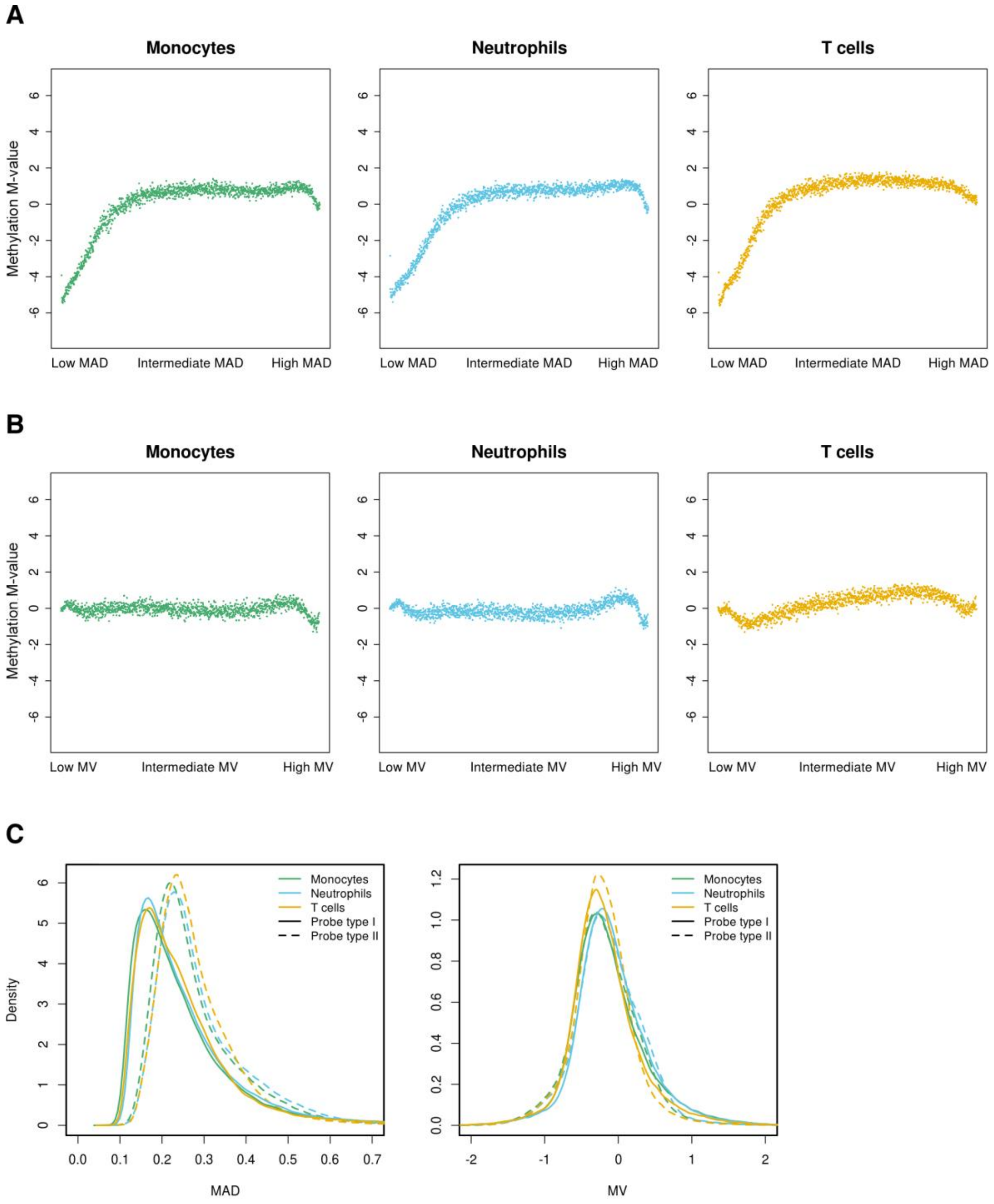

Figure S8. Robust quantification of inter-individual variability of DNA methylation levels. We compared two parameters that give account of DNA methylation variability, the MAD and DNA methylation variability value (MV). First, CpG-wise MAD-values were calculated. Then, these values were ordered from low to high MAD, grouped together in bins of 300 CpGs, and plotted against the mean DNA methylation M-values. The ordering by MAD-values was preserved to investigate whether the MAD-values are evenly distributed across mean DNA methylation M-values. The same was done for MV-values. (A) Mean M-values with regards to increasing MAD-values. We found that MAD-values are correlated with mean DNA methylation levels. (B) Mean M-values with regards to MV-values. In contrast to MAD-values, MV-values showed less dependence on the mean, enabling unbiased quantification of DNA methylation variability. (C) Distribution of MAD- and MV-scores in relation to 450K array probe types. While MAD-values exhibited differences in relation to Illumina probe type, MV-values did not.

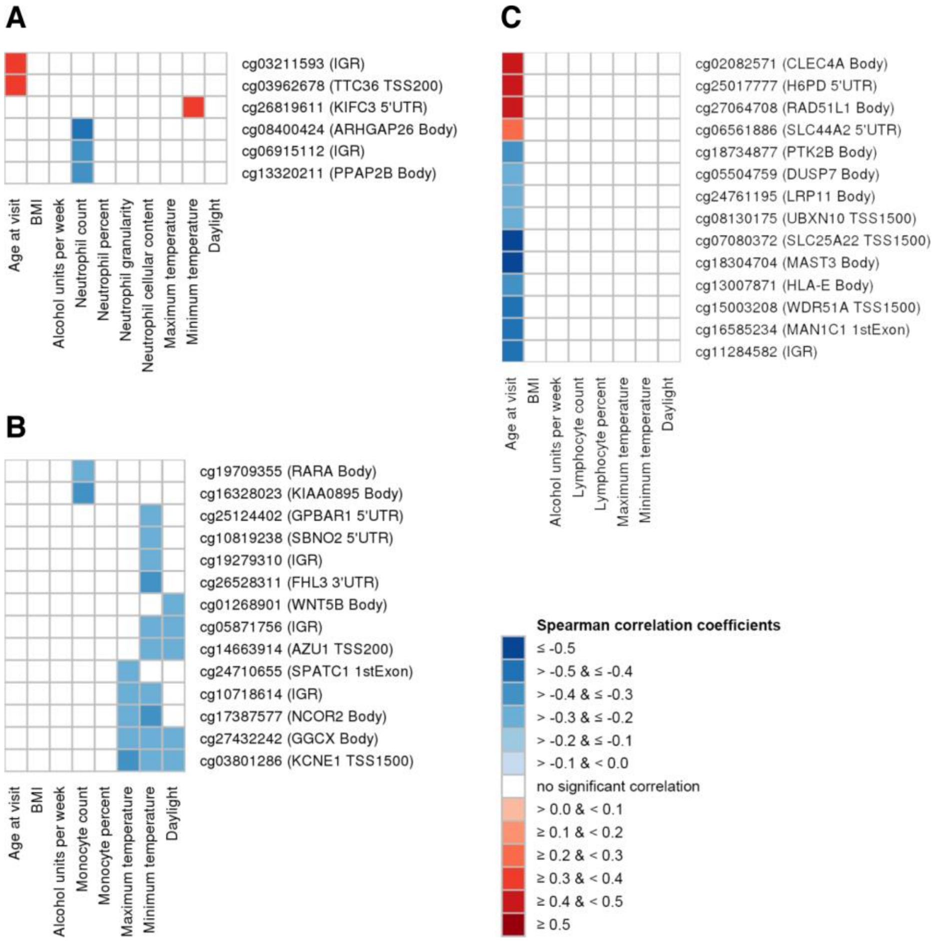

Figure S9. Correlation of cell type-specific hypervariable CpGs with donor information. Heatmap of Spearman's correlation coefficients showing cell type-specific HVPs that correlated with various quantitative traits in donors. Correlations are shown for (A) neutrophils, (B) monocytes, and (C) T cells. Only HVPs that correlated with at least one of the measured traits are shown (BH-corrected *P*-value <0.05, Spearman's rank correlation).

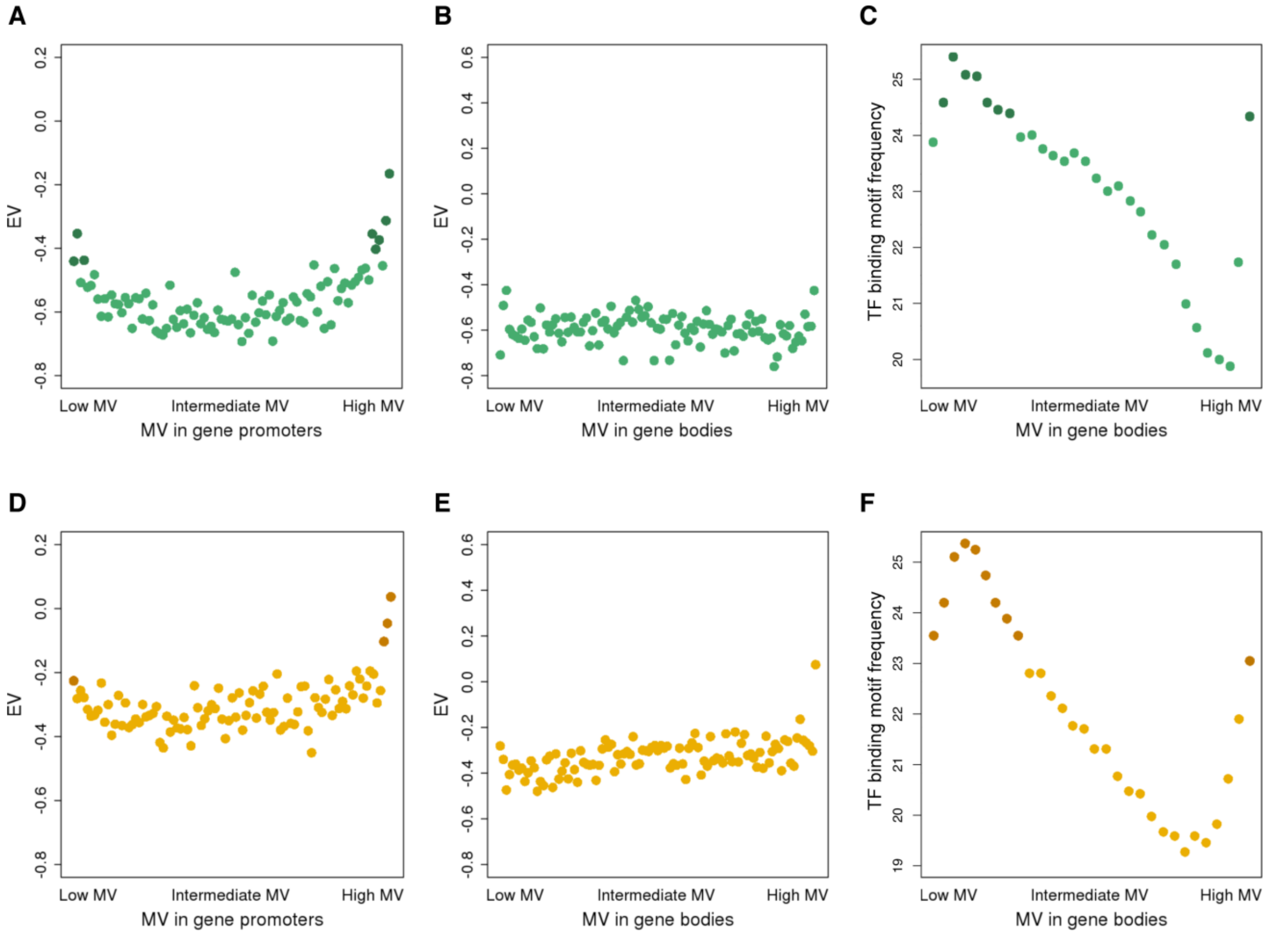

Figure S10. Global relationship between DNA methylation variability and gene expression variability at gene promoters and bodies. (A) Correlation between DNA methylation variability and gene expression variability at gene promoters in monocytes. First, gene-wise MV-values were calculated. Then, the values were ordered from low to high MV-value, grouped together in bins of 100 genes, and plotted against the EV-values, maintaining the ordering by MV-values. This binning strategy was applied to reduce the complexity of the data. HVPs at gene promoters were defined as CpG sites annotated to TSS1500, TSS200, 5'UTR, and first exon, according to the Illumina 450K array annotation manifest. Darker data points indicate the subset of bins that is further discussed in the Results section. (B) Same scatter plot as shown in panel (A) but for HVPs that map to gene bodies. HVPs at gene bodies were defined as CpGs annotated to body and 3'UTR, according to the 450K array annotation manifest. (C) Scatter plot of the number of consensus transcription factor binding motifs at promoter regions in monocytes versus MV-values. Promoter regions were defined as ±500 bp around the transcription start site. Darker data points indicate the subset of bins that is further discussed in the Results section. (D)–(F) Plots as shown in panels (A)–(C) but for T cells.

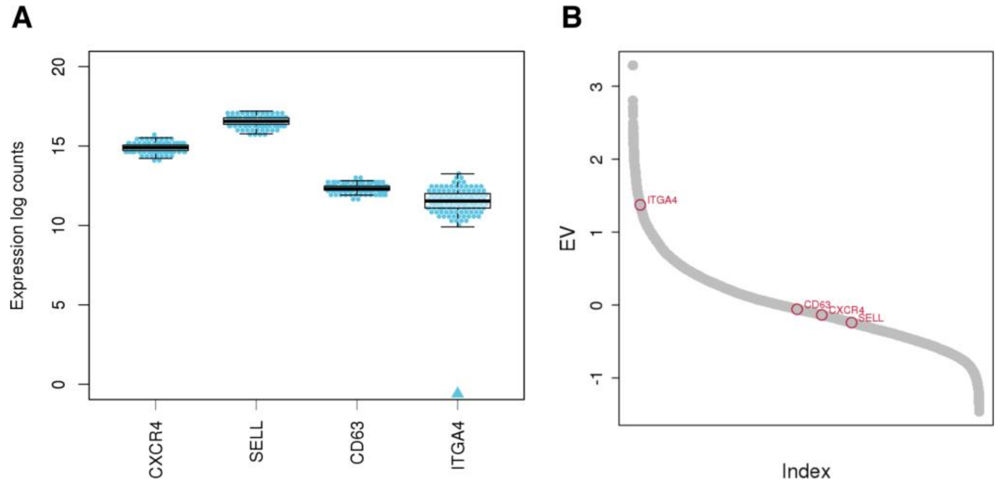

Figure S11. Gene expression of surface markers corresponding to known neutrophil subpopulations. We assessed the expression profiles of a number of genes that encode cell surface proteins indicating distinct cellular subpopulations of neutrophils. Differences attributed to such subpopulations can contribute to inter-individual heterogeneity. (A) Boxplots of gene expression values of cell surface markers in neutrophils. For each cell type, data points represent the expression values of the indicated gene in one individual. Cell types marked by an arrow were found to show significantly increased variability compared to monocytes and T cells. The markers were selected based on the literature (Silvestre-Roig et al. (2016), *Blood* 127(18), 2173-81): *CXCR4*, encoding chemokine (C-X-C motif) receptor 4, is upregulated in “aged” neutrophils. Mobilization of CD63 correlates with neutrophil elastase release, and represents a marker for cell activation status. *CD62L* (also known as *SELL*) encodes selectin L; its ligation and cross-linking results in neutrophil activation, including intracellular calcium release and superoxide production. *CD49* (also known as *ITGA4*), encodes integrin alpha-4 subunit 3, and is required for efficient inflammatory response against pathogen invasion. Note that the following previously characterized neutrophil subset markers were not present in our data set: *CD177/PRTN3*, *OLFM4*, *IL13*, *TCRA/TCRB*, and *IL17A*. (B) Distribution of the EV-values of all protein-coding genes assessed in our data set. The genes discussed in panel (A) are highlighted in red.
